# Anticipatory Behavior for a Mealworm Reward in Laying Hens Is Reduced by Opioid Receptor Antagonism but Not Standard Feed Intake

**DOI:** 10.3389/fnbeh.2019.00290

**Published:** 2020-01-14

**Authors:** Peta S. Taylor, Adam S. Hamlin, Tamsyn M. Crowley

**Affiliations:** ^1^Animal Science, School of Environmental and Rural Science, Faculty of Science, Agriculture, Business and Law, University of New England, Armidale, NSW, Australia; ^2^School of Science and Technology, Faculty of Science, Agriculture, Business and Law, University of New England, Armidale, NSW, Australia; ^3^iMPACT Institute School of Medicine, Deakin University, Waurn Ponds, VIC, Australia

**Keywords:** welfare, nalmefene, poultry, affective states, hedonic, pleasure, goal-directed, pharmacological

## Abstract

It is widely accepted that the absence of suffering no longer defines animal welfare and that positive affective experiences are imperative. For example, laying hens may be housed in environments that do not cause chronic stress but may lack particular resources that promote positive affective experiences, such as conspecifics or effective enrichment. Despite a consensus of how important positive affect is for animal welfare, they are difficult to identify objectively. There is a need for valid and reliable indicators of positive affect. Pharmacological interventions can be an effective method to provide insight into affective states and can assist with the investigation of novel indicators such as associated biomarkers. We aimed to validate a pharmacological intervention that blocks the subjective hedonistic phase associated with reward in laying hens *via* the administration of the non-selective (μ, δ, and κ) opioid receptor antagonist, nalmafene. We hypothesized that nonfood deprived, hens that did not experience a positive affective state when presented with a mealworm food reward due to the administration of nalmefene, would show minimal anticipatory and consummatory behavior when the same food reward was later presented. Hens (*n* = 80) were allocated to treatment groups, receiving either nalmefene or vehicle (0.9% saline) once or twice daily, for four consecutive days. An anticipatory test (AT) was performed on all days 30 min post-drug administration. Behavioral responses during the appetitive and consummatory phase were assessed on days 1, 3 and 4. Anticipatory behavior did not differ between treatment groups the first time hens were provided with mealworm food rewards. However, antagonism of opioid receptors reduced anticipatory and consummatory behavior on days 3 and 4. Feed intake of standard layer mash was not impacted by treatment, thus nalmefene reduced non-homeostatic food consumption but not homeostatic consumption. Behavioral observations during the AT provided no evidence that nalmefene treated hens were fearful, sedated or nauseous. The results suggest that we successfully blocked the hedonistic subjective component of reward in laying hens and provide evidence that this method could be used to investigate how hens perceive their environment and identify associated novel indicators to assess hen welfare.

## Introduction

Most ethologists accept that animals possess a number of basic affective states (emotions) that reflect the animals’ needs or wants (Fraser and Duncan, 1998; Duncan, 2002). Affective states are critical evolutionary adaptions; negative affective states protect animals and positive affective states reinforce low-cost behaviors that have positive long-term implications (Fraser and Duncan, 1998; Spruijt et al., 2001). Affective states include behavioral, autonomic and subjective components (Boissy et al., 2007). It has been argued that how an animal feels (i.e., the subjective component of affective states) largely contributes to, or defines, animal welfare (Désiré et al., 2002). However, the inability to directly assess the subjective component of emotions has historically slowed the progress of animal welfare science. Yet, if it is accepted that emotional states have evolved to motivate behavior, insight into an animal’s affective state may be inferred by assessing animal preferences, aversions and priorities. Indeed, various authors have reported indirect measures that infer affective states, particularly negative affective states such as pain and fear (Colpaert et al., 2001; Forkman et al., 2007). Positive affective states have received less attention, although it is widely accepted that good welfare is fundamentally the presence of positive experiences not simply the absence of the negative (Désiré et al., 2002; Boissy et al., 2007).

Advances in our understanding of the reward system have identified three key components; affect, motivation, and learning that constantly interact in reward processing (Berridge and Robinson, 2003). These three psychological components of reward processing; hedonic value (affect), wanting (motivation), and learning have discriminable neural mechanisms. These processes occur throughout the reward cycle, and while learning occurs throughout the cycle, wanting tends to dominate the initial appetitive phase, whereas hedonic pleasure dominates the consummatory phase (Berridge and Kringelbach, 2015). As such a rewarding experience effectively reduces a motivational state by moving an animal closer towards the desired state, by either pursuing a stimulus with high hedonic value or reducing a negative affective state (van der Harst and Spruijt, 2007). Generally, consummatory behaviors such as eating, drinking, sex and social interactions are considered rewarding for most animals (van der Harst and Spruijt, 2007). Additionally, an animal’s evolutionary history may result in species-specific goal-directed (rewarding) behaviors and stimuli, such as exploration or grooming (Spruijt et al., 2001). The continuously developing field of affective neuroscience is providing evidence of the relationships between neurophysiology and goal-directed behaviors.

The neurobiology of consummatory behaviors is dissociated from the neurobiology of appetitive behaviors (Berridge, 1996; Spruijt et al., 2001; Boissy et al., 2007; Berridge and Kringelbach, 2015), such that the *“liking”* consummatory phase is regulated by opioids and the *“wanting”* appetitive phase is regulated by dopamine (Berridge and Robinson, 1998; Boissy et al., 2007; Zimmerman et al., 2011; Berridge and Kringelbach, 2015). Mesolimbic dopamine levels are not synonymous with hedonia, but rather sensorimotor processes involved in motivation and responses to conditioned rewarding stimuli (Salamone et al., 1997; Kelley et al., 2002). Thus, dopamine is not directly involved in liking (Berridge and Robinson, 1998; Berridge and Kringelbach, 2015). However, opioids do affect *wanting*; as opioids act indirectly or directly to stimulate dopaminergic ventral tegmental area (VTA) neurons or by increasing dopamine release at the level of the nucleus accumbens (NAc; Burgdorf and Panksepp, 2006). Interactions between the aforementioned neuronal systems demonstrate the complexity of the reward system and highlight that the subjective experience of reward is not mediated by one specific brain region or neurotransmitter.

Behavioral indicators of motivation include anticipatory behavior which is a behavioral change, typically an increase in activity, that precedes a predictable event (Krebs et al., 2017). Positive anticipatory behavior is goal-directed and dependent on expectations or predictions, thus anticipatory behavior is reflective of the combination of genetics and ontogeny that shape the animals’ subjective experience (Spruijt et al., 2001). van der Harst and Spruijt (2007) provide evidence that anticipatory behavior is displayed when rats are given a conditioned cue announcing a reward (an enriched cage or sexual contact), but is absent when conditioned cues announce negative or neutral stimuli (barren cage, or cylinder filled with water). As such, anticipatory behavior is likely reflective of an appetitive positive affective state associated with dopaminergic activity in the brain (van der Harst and Spruijt, 2007; Moe et al., 2011).

Pharmacological interventions have been an important part of providing evidence of subjective experiences of reward in animals (Peciña, 1998). Administering opioid receptor agonists or antagonists can alter affective states and subsequently behavioral responses, including feeding and social behavior, play, and self-grooming (Boissy et al., 2007). For example, opioid agonists have shown to increase behavioral expressions associated with pleasure after consumption of highly palatable sucrose solutions (Doyle et al., 1993; Cagniard and Murphy, 2009) or hyperphagia specifically related to palatability and macronutrient content (Rockwood and Reid, 1982; Marks-Kaufman et al., 1984; Parker et al., 1992; Levine et al., 1995; Woolley et al., 2006; Le Merrer et al., 2009). Such methods of blocking or enhancing certain receptors can provide insight into how rewards, or other stimuli, are perceived by animals.

Nalmefene (17-[cyclopropylmethyl]-4,5α-epoxy-6-methylenemorphinan-3, 14-diol) is a pure opioid antagonist, which blocks μ, δ, and κ opioid receptors (Glass et al., 1994). Opioid receptors are expressed primarily in the cortex, limbic system, and brain stem, however, some structures have higher expression of one receptor over the others (Le Merrer et al., 2009). Additionally, specific hotspots in various areas of the brain have been identified, that when specifically targeted, result in different impacts on subjective experiences (Kelley et al., 2002; Castro and Berridge, 2014). For example, stimulation of μ-opioid receptors in the rostrodorsal hotspot in the medial shell of the NAc has shown to increase the hedonistic component of reward in rats, evidenced by increased orofacial reactions to sucrose with no impact on standard food intake (Castro and Berridge, 2014). Nalmefene is long-acting, potent and has a high affinity to central nervous system opiate receptors relative to other available opioid antagonists such as naloxone (Glass et al., 1994). Nalmefene targets all three opioid receptors across various brain structures such that both the motivational and hedonistic components of reward are likely impacted. Human studies show that feed intake is reduced after the administration of nalmefene despite no difference in self-reported hunger (Yeomans et al., 1990). Further investigation showed the nalmefene specifically reduced the intake of fat and proteins, not carbohydrates, suggesting that the differences in consumption were likely related to the palatability of the food rather than influences of hunger, i.e., how much the subject *liked* the food provided (Yeomans et al., 1990).

Positive emotions can be categorized into three temporal states; past (post-consummatory satisfaction), present (pleasant sensory activity) and future (expectations of positive affective states; Boissy et al., 2007). There has been much work on the future temporal state (motivation) in the field of animal welfare science, however minimal investigation into satisfaction. We attempted to alter post-consummatory contentment by blocking the *“liking”* response to a novel food reward with the non-specific opioid receptor antagonist, nalmefene and assessed the success of the intervention by measuring the expectation when presented with the same reward in the future. We hypothesized that if hens had not experienced hedonistic value (due to the administration of nalmefene) they would show no, or minimal, anticipatory behavior, hyperphagia of palatable mealworms and no difference in non-homeostatic food consumption.

Whilst there has been considerable investigation into the assessment of negative affective states in laying hens (Forkman et al., 2007) assessments of positive affective states are lacking. As such, researchers, industry and producers do not have the tools required to comprehensively assess hen welfare. Such assessments would enable measurable improvements in housing, enrichment programs, and industry benchmarking programs. The first step to identifying novel indicators of hen welfare specifically related to positive affective states is to validate a method that disrupts the pathway associated with a specific affective state, for example, the positive effect of liking associated with reward. Thus, we aimed to validate a pharmacological model to investigate the subjective experience of pleasure, as a step towards identifying novel indicators of positive affective states in laying hens.

## Animals, Materials and Methods

All animals and procedures used in this study were approved by the University of New England Animal Ethics Committee (Approval number 18-114).

### Animals and Housing

Eighty adult Hy-line hens of 80 weeks of age were sourced from a commercial conventional cage enterprise and transported to a research facility at the University of New England (Armidale, NSW, Australia). Hens were individually housed in cages (50 × 54 cm) but were not visually isolated from each other. Hens were fed a commercially available layer mash (Norco, South Lismore, NSW, Australia) *ad libitum* and water was available at all times *via* nipple drinkers. Feed intake over the treatment period was calculated for each individual hen. Hens were provided with natural ventilation and lighting *via* two curtains that were raised at approximately 6:00 h and lowered at approximately 19:00 h. but were also provided with artificial lighting on a 16:8 L:D schedule (4:00–20:00 h) as per the recommendations for Hy-line hens of 80 weeks from the breed management guide (Hy-Line International, 2018). Hens were habituated to the housing conditions, feed and research staff for 7 days.

### Treatments

Hens were randomly allocated to one of four treatment groups; nalmefene one dose daily (N1), nalmefene two doses daily (N2), saline one dose daily (C1) or saline two doses daily (C2). N1 and N2 hens received 0.5 ml of 0.4 mg/kg of nalmefene (17-[cyclopropylmethyl]-4, 5α-epoxy-6-methylenemorphinan-3,14-diol, nalmefene hydrochloride, 1B/220482, Tocris, Noble Park, Victoria, Australia) dissolved in 0.9% saline in the morning, administered *via* intramuscular injections into the pectoral muscle. The dose administered was based on the nalmefene dose-response study from Savory et al. (1989) on laying hen eating and drinking behavior. The half-life of nalmefene in humans is 12 h (Wang et al., 1998), but is unknown for hens. Therefore to determine if there were any negative welfare implications for hens dose with nalmefene every 12 h, e.g., theoretically keeping hens in a constant state of not experiencing reward the N2 treatments hens were dosed twice daily at 12-h intervals. N2 hens received an additional dose of 0.5 ml of nalmefene (0.2 mg/kg) 12 h after the morning injection. C2 hens were also dosed twice daily to control for any handling impacts. C1 and C2 hens received 0.5 ml 0.9% saline either once (C1) or twice (C2) daily at the same time and *via* same administration route as nalmafene treatment hens. Hens were dosed accordingly for four consecutive days.

### Anticipatory Behavior

**Figure 1:**
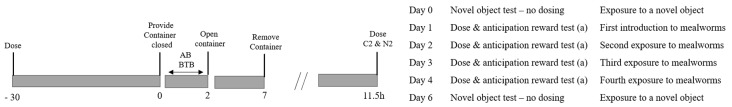
Experimental procedures including the time of dosing (−30 min) prior to the anticipatory test (AT) and behavioral time budget (BTB), subsequent dosing for C2 and N2 treatment hens and the procedures across consecutive days.

An anticipatory test (AT) was performed on all days from 1 to 4 (Figure 1), however, were only video recorded, and thus analyzed, on days 1, 3 and 4. Exactly 30 min after the morning injection and immediately after the feed was removed, five live mealworms in a transparent closed food container were presented at the front of their cage. The onset of action of nalmefene in hens was unknown so hens were tested based on the onset of action of nalmefene in humans after intramuscular administration, approximately 15 min (Dixon et al., 1986), with an additional 15 min lag to increase confidence in the likelihood that the nalmefene was altering hen state. Therefore, hens were tested 30 min after dosing. Hens could see the mealworms and could reach the container but could not access the mealworms due to the closed lid. Containers had two strips of green tape to provide a visual cue. After 1 min the lid was opened, and the hens were provided access to the mealworms, for a further 5 min. Visual contact with humans was restricted during the test excluding times when the mealworms were provided or the lid was removed and hens were visually isolated from other hens by placing a black curtain on either side of the cage. Hen behavior was recorded with a GoPro Hero 7 (GoPro, Carlsbad, CA, USA) for post-analysis. Latency to peck the container and number of pecks when the lid was closed was calculated, continuous observation for the whole 2 min, as an indicator of anticipation of the mealworm (reward). The number of pecks in the open container was quantified *via* continuous observations for the entire 5 min period as an indirect assessment of relative consumption.

Hen behavior (Table 1) was analyzed by one trained observer using instantaneous scan sampling (Altmann, 1974). Behavior was recorded every 15 s for 2 min when hens were provided the closed mealworm container. The proportion of observations for each behavior was calculated over the 2-min for each day of testing.

**Table 1:** Ethogram of hen behavior.

Behavior	Description
Interaction with container	Contact with container or head < 2 cm distance from the container.
Standing	Standing on feet, legs extended, no movement of the body with eyes open, head in a resting position (not outstretched).
Alert	Sitting or standing, with neck outstretched, head upright and eyes open.
Immobile	Frozen in a sitting or standing position, no head or body movement.
Walking	Upright position, legs are displaced and action of legs results in propulsive force.
Resting	Sitting on a cage wire floor, head at resting position (not outstretched) with eyes open or closed.
Preening	Grooming of plumage with the beak in either sitting or standing posture.
Pecking home cage	Pecking at the cage wiring.
Drinking	Beak in contact with nipple drinker or nipple drinker cup.
Other	Shake—body is moved side to side and feathers are ruffled, wing flap—up and down movement of both wings in a standing position, wing stretch—wings are stretched backward and held behind the birds for at least 1 s with no flapping, scratch—feet are used to scratch any surface of the cage.

### Response to Novelty

Response to novelty was tested pre-treatment (day 1) and post-treatment (day 6) by placing a novel object (NO; pre-treatment: pink dog chew toy (20 cm × 4 cm); post-treatment: spiked yellow ball (10 cm diameter). Hens were tested in their home cages to avoid any handling effects. Hens were visually isolated from other hens by placing a black curtain on either side of the cage. NOs were placed on top of the hens’ feed tray and left for 10 min. Latency to peck the NO was assessed as an indicator of neophobia and exploration.

### Statistics

All statistical analysis was performed with SPSS statistical software (v22, IBM Corporation, Armonk, NY, USA). Comparisons between treatments for the percentage of hens that pecked the open or closed box were analyzed with a Chi-Square model, multiple comparisons were corrected with the Bonferroni method. Censored data, including latency to peck the container or NO, were analyzed with Kaplan–Meier survival analysis and compared treatments on each test day. Handling treatment groups (i.e., the treatment groups that were handled and dosed twice daily; C2 and N2) within drug treatment (control or nalmefene) did not differ in latency to peck the closed container (*p* > 0.05) therefore these data were pooled to compare results within treatment group (control or nalmefene) across days. The data for pecks on the closed and open container did not meet the criteria for normality and therefore were analyzed with a non-parametric generalized linear model with a Poisson distribution with a log link function. Treatment, day and the interaction between treatment and day were included in the model as fixed factors. *Post hoc* comparisons were corrected with the least-squares difference method to account for multiple comparisons.

Behavioral Time Budget (BTB) data did not meet the criteria for normality, despite transformation attempts. Therefore, non-parametric Mann–Whitney comparisons were used to compare the effect of handling within treatment (e.g., control once daily compared to control twice daily) for each testing day. There were no differences between handling groups within treatment therefore data were pooled within treatment and comparisons between control and nalmefene treated hens for each testing day were compared using a two-tailed Mann–Whitney analysis. Feed intake data met the criteria for normality and was analyzed with an ANOVA comparing treatment groups.

## Results

There was no difference in the proportion of hens that pecked the closed mealworm container between treatment groups on day 1 ($\chi_{\left(3,36\right)}^{2}$ = 5.22, *p* = 0.156; Table 2). More C2 hens pecked the closed mealworm container than hens from both of the nalmefene treatment groups (N1 and N2) on day 3 ($\chi_{\left(3,36\right)}^{2}$ = 17.1, *p* = 0.001; Table 2). More hens from both control groups (C1 and C2) pecked the closed mealworm container on day 4 than both nalmefene treatment groups ($\chi_{\left(3,36\right)}^{2}$ = 24.4, *p* < 0.001; Table 2).

**Table 2:** Hen responses to the presentation of a closed transparent container containing live mealworms, including the percentage of hens within each treatment that pecked the closed container over time and latency (seconds) to peck the open container.

	Pecked closed container (% hens)				Latency to peck closed container (s)			
	C1	C2	N1	N2	C1	C2	N1	N2
Day 1	50.0	40.0	12.5	10.0	95.7 ± 13_a_	94.5 ± 12_a_	108.1 ± 12_b_	108.0 ± 12_b_
Day 3	66.7_a, b_	88.9_b_	12.5_a_	10.0_a_	51.2 ± 18_a_	33.3 ± 15_a_	105.9 ± 14_b_	113.4 ± 7_b_
Day 4	90.0_a_	100.0_a_	22.2_b_	10.0_b_	28.9 ± 14_a_	6.8 ± 2_b_	95.9 ± 16_c_	111.5 ± 9_c_

*Hens received either a dose of saline (C1 and C2) or nalmefene (N1 and N2) 30 min prior to testing on day 1, 3 or day 4. Hens were dosed either once (C1 and N1) or twice (C2 and N2) daily. Letters with differing subscript indicate significant differences between treatments within a row at *p* < 0.05*.

**Figure 2:**
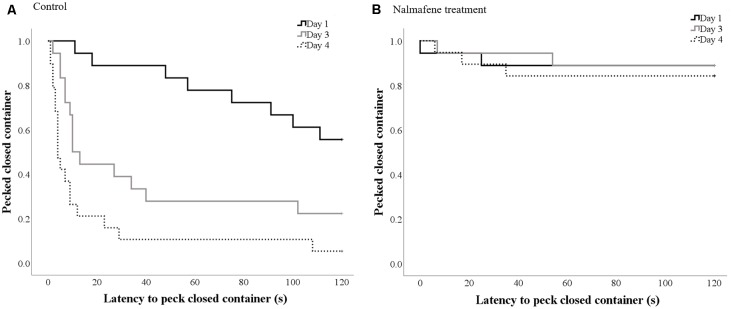
Kaplan-Meier curves indicating the proportion of control **(A)** and treatment **(B)** hens that pecked the closed container (y-axis) over time (seconds; x-axis) on each day of dosing/testing (day 1 solid black line; day 3 solid gray line; day 4 dotted gray line). Every time a hen-pecked the container, the probability on the y-axis drops.

Control hens were quicker to peck the closed container over time ($\chi_{\left(1,39\right)}^{2}$ = 39.3, *p* < 0.001; Figure 2A). The latency to peck the closed container over time did not differ for hens dosed with namelfene (*p* = 0.35; Figure 2B).

Hens from the control groups were quicker to peck the closed mealworm container on all days than hens that received nalmefene (day 1: $\chi_{\left(1,39\right)}^{2}$ = 4.3, *p* = 0.038; day 3: $\chi_{\left(1,39\right)}^{2}$ = 17.15, *p* < 0.001; day 4: $\chi_{\left(1,39\right)}^{2}$ = 29.8, *p* < 0.001; Table 2).

**Figure 3:**
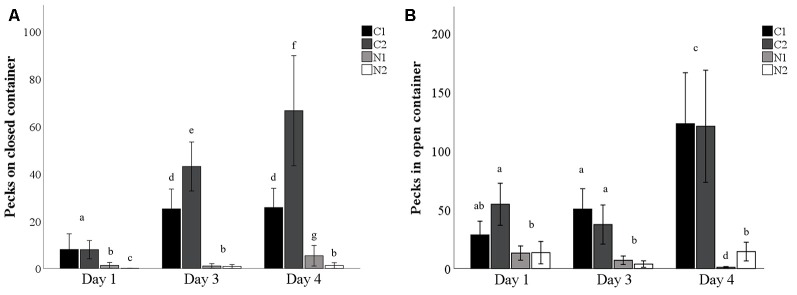
The number of pecks on a closed **(A)** and open **(B)** transparent container containing mealworms when presented to hens on day 1, 3 and 4 dosing either once (C1, N1) or twice (C2, N2) daily with saline (C1–black bar, C2–dark gray bar) or nalmefene (N1–light gray bar, N2–white bar) hens were presented with the container 30 min after dosing. Bars with differing subscript indicate significant differences between treatments and day of testing (*p* < 0.05).

The average number of pecks on the closed mealworm container increased over time (testing/dosing days), particularly for the control hens that were injected with saline twice daily ($\chi_{\left(1,98\right)}^{2}$ = 7.7, *p* = 0.005; Figure 3). Although treatment hens pecked the closed container slightly more over time, they pecked the closed container fewer times than the control hens at all time points ($\chi_{\left(8,148\right)}^{2}$ = 153.0, *p* < 0.001; Figure 3). Hens that were dosed with nalmefene twice daily pecked the closed container less than hens that were dosed with nalmefene only once a day on day 1 and 4 (*p* ≤ 0.02; Figure 3). Conversely, hens that were dosed twice daily with saline pecked at the closed container more than hens dosed once daily with saline on day 3 and day 4 (both *p* < 0.001; Figure 3).

The proportion of hens that pecked the open mealworm container did not change over time (*p* = 0.397; Table 3). More control hens pecked the open mealworm container than nalmefene treatment hens on day three ($\chi_{\left(3,148\right)}^{2}$ = 21.7, *p* < 0.001; Table 3). Control hens were quicker to peck the open container than hens from the nalmefene treatment group on all days (day 1: $\chi_{\left(1,39\right)}^{2}$ = 5.9, *p* = 0.015; day 3: $\chi_{\left(1,39\right)}^{2}$ = 12.9, *p* < 0.001; day 4: $\chi_{\left(1,39\right)}^{2}$ = 18.2, *p* < 0.001; Table 3). Control hens were quicker to peck the open container over time ($\chi_{\left(1,39\right)}^{2}$ = 39.3, *p* < 0.001; Table 3) there was no difference over time for nalmafene treatment hens (*p* = 0.35; Table 3). There was no interaction between treatment and day on the number of pecks in the open container (*p* = 0.645). However on all days, control hens pecked in the open container more than nalmefene treatment hens (*F*_(3,95)_ = 6.6, *p* < 0.001; Figure 3).

**Table 3:** Hen responses to the presentation of an open transparent container containing live mealworms, including the percentage of hens within each treatment that pecked the open container and latency (seconds) to peck the open container.

	Pecked open container (% hens)					Latency to peck open container (s)			
	C1	C2	N1	N2	*p*-value	C1	C2	N1	N2
Day 1	75.0	90.0	50.0	40.0	0.09	104.4 ± 44_a_	73.7 ± 33_a_	174.4 ± 49_b_	191.2 ± 45_b_
Day 3	77.8_a, b_	100.0_b_	50.0_a, b_	30.0_a_	0.01	68.9 ± 44_a_	33.4 ± 29_a_	155.4 ± 29_b_	235.1 ± 39_c_
Day 4	90.0	100.0	55.6	60.0	0.06	55.2 ± 36_a_	2.11 ± 1_b_	174.1 ± 46_c_	174.1 ± 40_c_

*Hens received either a dose of saline (C1 and C2) or nalmefene (N1 and N2) 30 min prior to testing on day 1, 3 or day 4. Hens were dosed either once (C1 and N1) daily or twice (C2 and N2). Letters with differing subscript indicate significant differences between treatments within a row at *p* < 0.05*.

### Behavioral Time Budgets

When presented with a closed mealworm container for 2 min on day 1, hens spent most of the time alert (control 60.5 ± 5.6%; nalmefene hens 75.3 ± 6.0%; Table 4). However, control hens spent more time interacting with the container (day 1: *z*_(2,36)_ = −2.7, *p* = 0.037; day 3: *z*_(2,36)_ = −4.2, *p* < 0.001; day 4: *z*_(2,36)_ = −4.5, *p* < 0.001) and more time standing than treatment hens on day 3 (*z*_(2,36)_ = −2.9, *p* = 0.005) and day 4 (*z*_(2,36)_ = −3.9, *p* < 0.001). There was no difference in time spent interacting with the container between treatment groups on day 1 (*p* = 0.790). Hens from both treatment groups were rarely immobile or resting during the test on any day (Table 4).

**Table 4:** Behavioral time budgets for hens administered with saline (control) or an opioid antagonist (nalmefene) after a closed container with five mealworms was presented in the home cage for 2 min.

Behavior	Treatment	Day 1	Day 3	Day 4
Interaction with container	Control	16.0 ± 5.8	0.6 ± 0.6	43.2 ± 7.5^a^
	Nalmefene	1.9 ± 1.3^b^	48.9 ± 6.8^a^	6.2 ± 4.0^b^
Standing	Control	12.3 ± 4.4	11.1 ± 3.9	9.9 ± 2.7^a^
	Nalmefene	34.0 ± 6.7^b^	10.5 ± 3.2^a^	36.0 ± 4.4^b^
Alert	Control	60.5 ± 5.6	75.3 ± 6.0	32.1 ± 5.5^a^
	Nalmefene	52.5 ± 6.8^b^	31.7 ± 5.2	38.0 ± 4.7
Immobile	Control	0.0 ± 0.0	0.0 ± 0.0	0.0 ± 0.0
	Nalmefene	5.5 ± 4.4	0.0 ± 0.0	0.0 ± 0.0
Walking	Control	3.1 ± 1.2	1.9 ± 1.0	4.9 ± 1.6
	Nalmefene	4.3 ± 2.5	2.5 ± 1.2	6.8 ± 1.6
Resting	Control	0.6 ± 0.6	1.2 ± 1.2	4.9 ± 4.9
	Nalmefene	4.3 ± 4.3	0.0 ± 0.0	0.6 ± 0.6
Preening	Control	1.9 ± 1.3	0.0 ± 0.0	0.0 ± 0.0
	Nalmefene	1.9 ± 1.3	5.0 ± 3.7	4.9 ± 4.9
Pecking home cage	Control	5.6 ± 1.9	3.7 ± 2.0	4.9 ± 3.2
	Nalmefene	0.6 ± 0.6	1.3 ± 0.9	7.4 ± 3.3
Drinking	Control	0.0 ± 0.0	0.0 ± 0.0	0.0 ± 0.0
	Nalmefene	0.0 ± 0.0	0.6 ± 0.6	0.0 ± 0.0
Other	Control	0.0 ± 0.0	0.0 ± 0.0	0.0 ± 0.0
	Nalmefene	0.6 ± 0.6	0.0 ± 0.0	0.0 ± 0.0

*Differing superscripts indicate a significant difference (*p* < 0.05) between treatments within a day of dosing*.

### Feed Intake

There was no statistical difference in feed intake over the trial period between any treatment groups, although numerically hens dosed twice daily with nalmefene consumed less than the other three treatment groups (Average feed intake: C1 281.2 ± 63.2 g; C2 217.6 ± 67.4 g; N1 229.7 ± 53.6 g; N2 83.2 ± 38.9 g; *p* = 0.114).

### Response to Novelty

**Figure 4:**
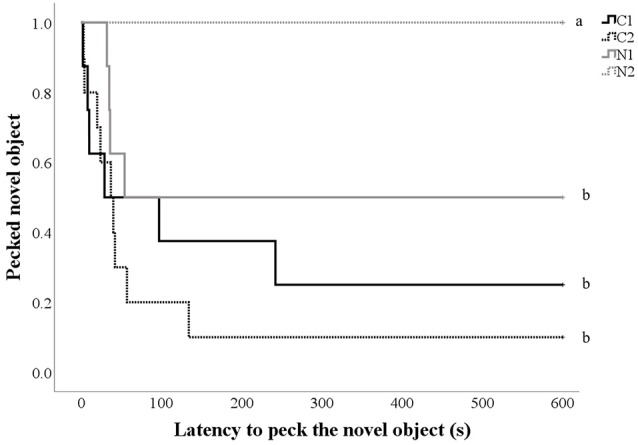
Kaplan-Meier curves indicating the proportion of control and treatment hens that pecked the novel object (NO; yellow spiked ball; y-axis) over time (seconds; x-axis) post-treatment. Every time a hen-pecked the NO, the probability on the y-axis drops. Hens received either a dose of saline (C1 and C2) or nalmefene (N1 and N2) for four consecutive days before the NO test was conducted, without the administration of any drug. Hens were dosed either once daily (C1 and N1) or twice daily (C2 and N2). The differing subscript is indicative of differences between treatment groups *p* < 0.05.

Only two hens touched the NO during the pre-treatment NO test, and there was no impact of treatment (*p* = 0.60). However post-treatment, control hens and hens that received nalmefene once daily were more likely to touch the NO (hens that touched the NO: C1 38.9% *n* = 7; C2 50.0%, *n* = 10; N1 23.5%, *n* = 4; N2 0.0%, *n* = 0; Figure 4) and to do so more quickly than hens that received nalmefene twice daily ($\chi_{\left(3,36\right)}^{2}$ = 16.9, *p* = 0.001; Figure 4).

## Discussion

We aimed to induce reward expectancy in hens by providing visual access to a mealworm container consecutively over 4 days. We expected to see indicators of anticipation in the hens that were expecting a reward. However, if hens had not experienced a positive affect associated with reward during previous experiences with mealworms (e.g., they did not *like* the mealworm due to blocked opioid receptors) such hens would show no indication that they expected (*wanted*) a reward (Berridge, 1996; Spruijt et al., 2001). Our results indicate that hens that received the non-specific opioid antagonist did not experience reward, as indicated by a reduction in anticipatory behavior when the visual cue was presented for 2 min before access to mealworms was provided and a reduction in the number of pecks to the open mealworm container, an indirect assessment of consumption, and no difference in homeostatic food consumption of standard layer mash. However, we take caution in the interpretation of these results as reward pathways are complex and off-target effects of nalmefene in laying hens are unknown.

Previous work has shown that sensitization of the reward system can be achieved *via* experiencing highly rewarding stimuli (Boissy et al., 2007). However, stressful experiences such as barren captive housing can also sensitize the reward system. For example, van der Harst et al. (2003a,b) provide evidence that rats housed in barren environments expressed more anticipatory behavior than rats kept in enriched environments. Conversely, chronically stressed animals often show symptoms of anhedonia and lose the ability to anticipate (van der Harst and Spruijt, 2007). As all of the hens in the current study were sourced from the same farm, housed in the same conditions throughout their early life and during the experiment, and no indicators of chronic stress were observed (e.g., abnormal behaviors or immobility) is it unlikely that negative previous experiences had any impact on the results.

Live mealworms were provided in the current study as these are preferred by hens over other foods, including wheat, cheese and sugary sweets (Bruce et al., 2003). Furthermore, hens will work for mealworms even if fully satiated (Moe et al., 2013), suggesting that hens find mealworms rewarding. We provide evidence that hens in the current study found mealworms rewarding as control hens approached the closed and open container faster from the 1st to the 4th day of testing. Consumption of food may be separated into homeostatic and non-homeostatic, either providing necessary nutritional uptake to sustain life or consumption driven by processes such as hedonics, respectively. Non-homeostatic consummatory behavior often leads to overconsumption (Olszewski and Levine, 2007).

Opioids play a central role in the hedonic evaluation of foods (Le Merrer et al., 2009) but also contribute to the incentive motivation to consume food, drugs and other rewards (Zhang et al., 2003; Grueter et al., 2012). We did not target specific opioid receptors, nor specific areas of the brain, therefore we likely impacted both the hedonistic and motivational component of the reward system. We do provide some evidence that we were able to disrupt the hedonistic component of reward, evident by a difference in mealworm consumption, albeit indirectly assessed, and no difference in the homeostatic consumption behavior of their standard layer mash feed over the course of the experiment. This approach of assessing homeostatic and non-homeostatic consummatory behavior, has been used with rats to identify specific hotspots in the brain that regulate the hedonistic component of reward (Rockwood and Reid, 1982; Marks-Kaufman et al., 1984; Levine et al., 1995; Castro and Berridge, 2014) For example, Castro and Berridge (2014) disassociated motivation to eat compared to sensory pleasure by stimulating specific hotspots in the NAc in rats which subsequently increased consumption of M&M candies but not standard rat chow. Additional methods that have aimed to separate the liking and wanting component of reward have used facial expressions, specifically orofacial responses to sweet or fatty food rewards in rats, humans and nonhuman primates (Berridge, 2000; Peciña and Berridge, 2005). Although, providing great insight into the subjective hedonic experience this method has not been validated, nor would be feasible, in particular species such as hens. Thus, there is a need to further develop methodology suitable for hens to differentiate the hedonistic and motivational component of reward, the anticipatory behavior reported in this study cannot provide such insight conclusively. Future investigations should consider assessing hen anticipatory behavior on subsequent days without the administration of nalmefene.

Whilst we found no significant difference in standard feed consumption between our treatment groups, N2 feed intake was numerically lower than the other treatment groups which are in agreement with Savory et al. (1989). The dose rates used in the current study were based on the Savory et al. (1989) study that provided evidence that feed intake was reduced at the dose rate of 0.4 mg/kg. The differences in methodological designs make it difficult to identify the cause of discrepancy between the two studies but may include differences in diet composition or the short term assessments of feed intake over 7 h post drug administration in the Savory et al. (1989) study compared to the total feed intake assessed over 4 days in the current study. Additionally, there were inconsistencies in the Savory et al. (1989) study, such that feed intake only statistically differed in the 1st, 3rd and 5th-h post-drug administration, but not the second or seventh. A better understanding of the impact of nalmefene on homeostatic and non-homoeostatic food consumption is required which may include both short and long term assessments of feeds that differ in palatability.

*Wanting* was assessed in the current study by behavioral indicators of anticipatory behavior. However, there was no evidence of anticipatory behavior as defined by Moe et al. (2009) “*hens standing alert with head and neck stretched*.” However, we did observe an increase in activity, similar to the increased frequency of head movements previously observed in laying hens (Zimmerman et al., 2011; Moe et al., 2013) and an increased focus towards the site where the reward was offered which is in agreement with anticipatory behavior in pigs (Petherick and Rutter, 1990), mink (Hansen and Jeppesen, 2004) and silver foxes (Moe et al., 2004). Further evidence of *wanting* was observed as control hens pecked the closed container more frequently which is likely indicative of motivation to access the reward. Such examples of increased “work” to access a reward has previously been observed in various laying hen operant conditioning trials (Lagadic and Faure, 1987; Olsson and Keeling, 2002). As anticipatory behavior is thought to reflect the animal’s perception of a stimulus we interpret the hen’s behavior to be an anticipation of the mealworm reward and conclude that control hens found the mealworms more rewarding than hens that were administered nalmefene. However, it must be noted that motivation (Le Merrer et al., 2009) or learning (Clissold and Pratt, 2014) may have also been directly impacted by nalmefene administration due to the off-target action of the drug on all opioid receptors.

We provide evidence that the administration of nalmefene (once or twice daily) successfully disrupted the opioid-mediated reward pathway in laying hens, in contrast to previous studies that used naloxone (Moe et al., 2013). Although previous work has shown that blocking opioid receptors with naloxone decrease the frequency of anticipatory behaviors in rodents (Spruijt et al., 2001; Woolley et al., 2006), it may not be as effective on hens. Indeed, previous research has shown that nalmefene was more effective in reducing feed intake in broiler chickens than naloxone (Savory et al., 1989). Additionally, naloxone has shown to have minimal effects on the behavior of other species including sheep (Verbeek et al., 2012) cattle (Rushen et al., 1999) and quail (Kostal and Kohutova, 2013).

Latency to peck the open mealworm container decreased over consecutive days of testing for control hens, but not treatment hens. Hens that received nalmefene waited more than 2 min, on average, before pecking at the open container and whilst this result may reflect a difference in motivation to consume the mealworms, it may simply be explained by an attraction to the live mealworm movement or impacts of satiety or the disruption of all opioid receptors rather than specific μ-opioid receptors that appear to regulate the hedonistic component of reward (Kelley et al., 2002; Castro and Berridge, 2014). However, differences in consumption of the mealworms could also be explained by unknown side effects of nalmefene administration which may include increased fearfulness, nausea, sedation or reduced curiosity. Indeed, various systems have been disrupted when specific opioid receptors are knocked out in mice; specifically motivation and anticipatory behavior in μ-receptor knockout mice (Kas et al., 2004), anxiety and depression in δ-receptor knockout mice (Filliol et al., 2000) or hallucinogenic activity when κ-opioid agonist are administered in mice (Yan and Roth, 2004). It is unlikely that nalmefene hens in the current study were sedated, as no differences in alertness or resting was observed between treatment and control hens. The NO test did suggest that nalmefene treated hens were more neophobic post-treatment than control hens, however this result may not be indicative of fearfulness. Forkman et al. (2007) suggest that the NO test is reflective of both inspective curiosity *and* neophobia (fearfulness). As there was no difference in immobility between treatment and control hens during the AT which is a typical behavioral indicator of fear in laying hens (Forkman et al., 2007), we suggest that the differences observed in the post-treatment NO test are more likely indicative of inspective curiosity. Although we cannot rule out that a reduction in motivation to explore in nalmefene treated hens was related to nausea induced by the drug (Cramer and Stanton, 2015), the NO test was performed when hens were not under the influence of the drug suggesting that previous experience and disruptions to the reward system may have had non-specific impacts on expectancy, exploration and other appetitive activities (Panksepp, 2005) or mood (Mendl et al., 2010). Further investigations are required and should consider assessing the impact of accessing non-food rewards to reduce the potential impact of homeostatic food motivations, or side effects from nalmefene that may have reduced the impact of motivation to eat.

Administration of either saline or nalmefene twice daily had an impact on anticipatory behavior compared to hens that were only dosed once daily. Rather than a nalmefene dose-response, this was likely an impact of handling as this effect was present in both nalmefene treated and control hens. Hens that were handled twice daily were likely less fearful of humans. These results likely reflect differences in human avoidance behavior when containers were delivered, or opened, by human experimenters, subsequently impacting latency to peck data and time available to interact with the container. This result serves as an important reminder of the impact of human researchers on subjects and the need to control for such impacts in experimental designs.

Hens treated with an opioid antagonist, nalmefene, showed less anticipatory behavior when a visual cue announcing the arrival of live mealworms was presented. Furthermore, nalmefene administration reduced non-homeostatic food consumption but not homeostatic consumption. Our results suggest that we successfully blocked the hedonistic subjective component of reward in laying hens and provides evidence that nalmefene could be used to further investigate how hens perceive their environment, specifically related to pleasurable experiences. However, nalmefene may have additionally, directly impacted the motivation to access food reward and further investigation into the impact of nalmefene on the liking component of reward compared to the wanting component warrants further investigation. As positive affective states are a critical aspect of animal welfare, understanding which environments and resources yield such experiences to hens will assist to develop science-based evidence to inform decisions on housing and effective enrichment programs for laying hens in commercial egg industries. Furthermore, the administration of nalmafene in laying hens may assist to identify associated novel indicators which can be implemented in on-farm welfare assessment programs to benchmark and safeguard hen welfare in a variety of contexts.

## Data Availability Statement

The datasets generated for this study are available on request to the corresponding author.

## Ethics Statement

The animal study was reviewed and approved by University of New England Animal Ethics Committee; Approval number 18-114.

## Author Contributions

PT wrote the manuscript and conducted all analysis. PT and TC conducted the experiment. All authors helped with developing the concepts, methodologies, conceiving the experiments and contributed to manuscript writing.

## Conflict of Interest

The authors declare that the research was conducted in the absence of any commercial or financial relationships that could be construed as a potential conflict of interest.
